# Supplementary material for: Indirect genomic effects shape cancer risk across species
Source: bioRxiv. 2026 Jun 29:2026.06.29.735167. Preprint. [Version 1] doi: 10.64898/2026.06.29.735167 (PMC13345119; doi:10.64898/2026.06.29.735167)
Supplement: Supplement 3 [file NIHPP2026.06.29.735167v1-supplement-3.pdf]

bioRxiv preprint doi: https://doi.org/10.64898/2026.06.29.735167; this version posted June 30, 2026. The copyright holder for this preprint (which was not certified by peer review) is the author/funder, who has granted bioRxiv a license to display the preprint in perpetuity. It is made available under a CC-BY 4.0 International license.

Supplementary Figure Legends

Supplementary Figure 1: Assembly contiguity and gene conservation in Birds and Mammals. A) Distribution of scaffold N50 values for Birds (green) and Mammals (blue). **B**) Distribution of average gene identity scores for genes lifted from human using Miniprot. Identity scores represent the proportion of aligned amino acid residues that are identical between the lifted ortholog and the human reference sequence. **C**) Distribution of average positive substitution scores for genes lifted from human using Miniprot. Positive substitution scores represent the proportion of aligned residues that are identical or biochemically similar (conservative substitutions).

Supplementary Figure 2: Model performance. In all cases, multiple sequence alignments (MSAs) containing birds and mammals are shown in green and mammals only are shown in blue. **A**) A histogram of gene size, measured as the number of nucleotides, stratified by the breadth of species in the alignment. **B**) A plot of the log-likelihood difference between fitting a single rate class per branch with no site-to-site variation compared to a full adaptive rate class model. The log-likelihood difference is positively associated with gene size in MSAs containing birds and mammals ($p < 0.001$) and mammals only ($p < 0.001$). **C-D**) Histograms of the marginal R^2 values stratified by the number of classes for **C**) benign and **D**) malignant tumour prevalence (see Methods).

Supplementary Figure 3: Evaluating the effect of alignment errors. Estimated dN and dS effect sizes on benign or malignant tumour prevalence with and without BUSTED-E filtering. **A-B**) Genes with a significant positive or negative association between dN or dS and benign tumour prevalence with and without BUSTED-E filtering are shown in red and blue respectively. **C-D**) Genes with a significant positive or negative association between dN or dS and malignant tumour prevalence with and without BUSTED-E filtering are shown in orange and green respectively. In all cases, genes with a significant dN or dS effect with and without BUSTED-E filtering but opposing directionality are shown in grey (see Methods). The strong concordance between effect size estimates before and after filtering indicates that the primary results are robust to potential alignment errors.

Supplementary Figure 4: Controlling for species longevity. A-**D**) Comparison of significant genes identified using the full model and secondary model that included species longevity as an additional covariate (see Methods). Qualitatively similar patterns were observed between models with and without longevity included as an additional covariate, 'longevity model' and 'full model' respectively. Numbers indicate genes identified exclusively by the full model (green), exclusively by the longevity model (purple), or shared between both models (grey). The substantial overlap between models indicates that primary results are robust to the inclusion of species longevity.

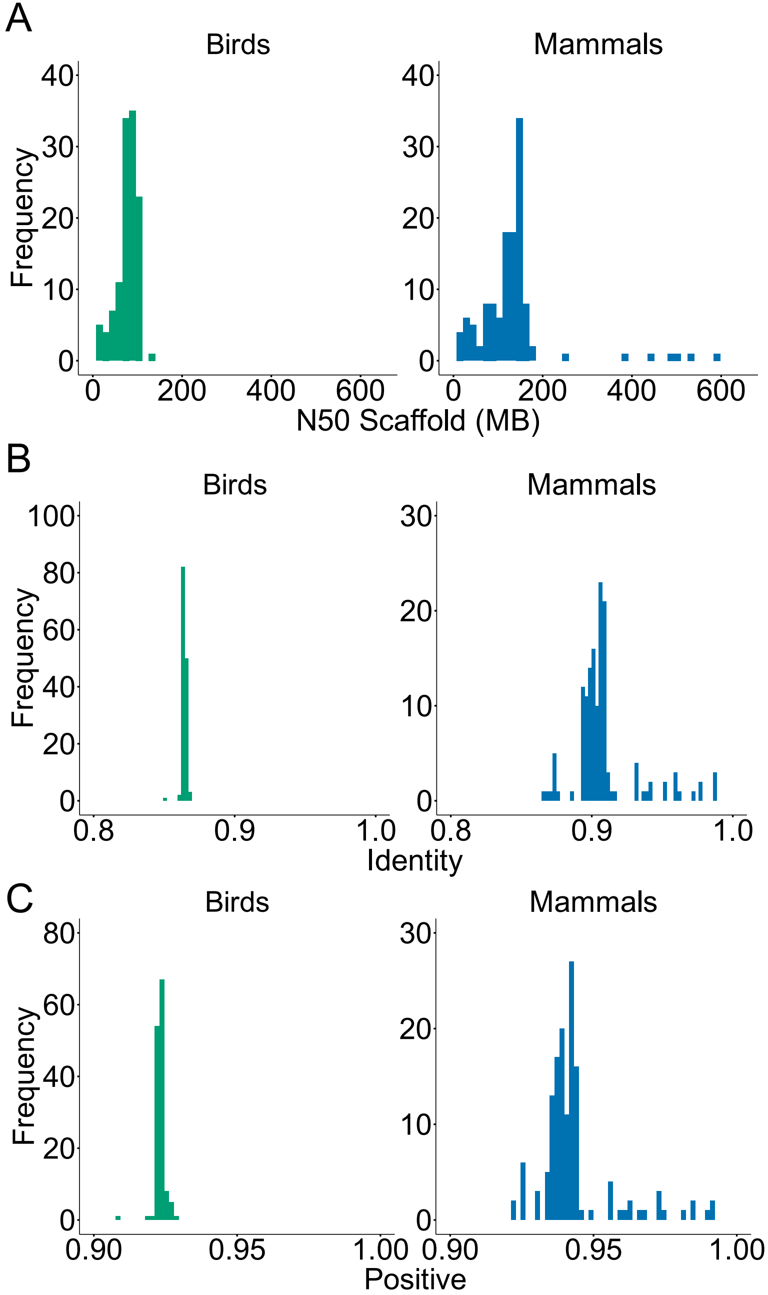

A
Birds
Mammals
Frequency
0
10
20
30
40
0
200
400
600
N50 Scaffold (MB)
B
Birds
Mammals
Frequency
0
20
40
60
80
100
0
10
20
30
0.8
0.9
1.0
Identity
C
Birds
Mammals
Frequency
0
20
40
60
80
0
10
20
30
0.90
0.95
1.00
Positive

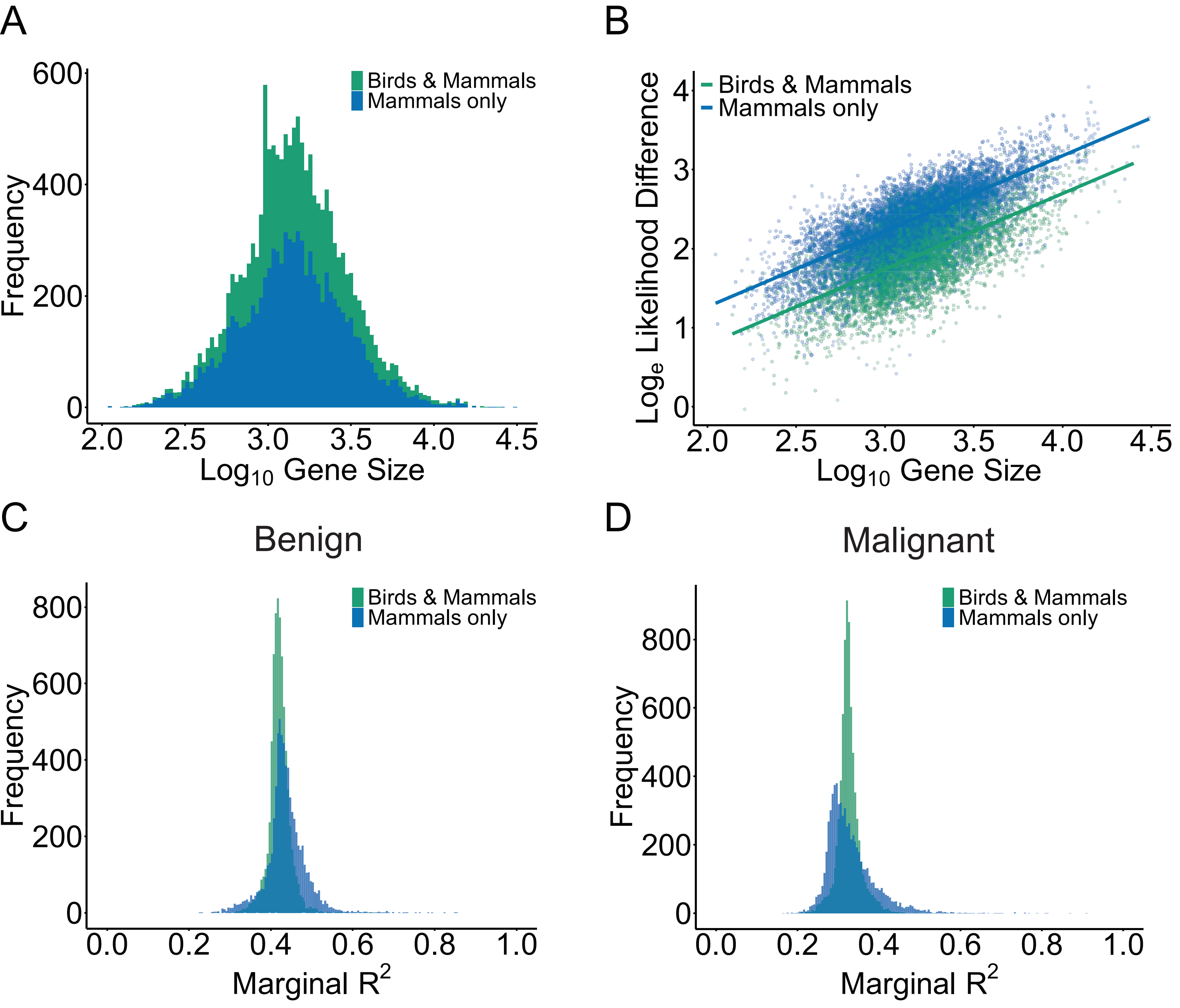
A
B
C
D
Birds & Mammals
Mammals only
Frequency
Log10 Gene Size
Loge Likelihood Difference
Benign
Malignant
Marginal R2

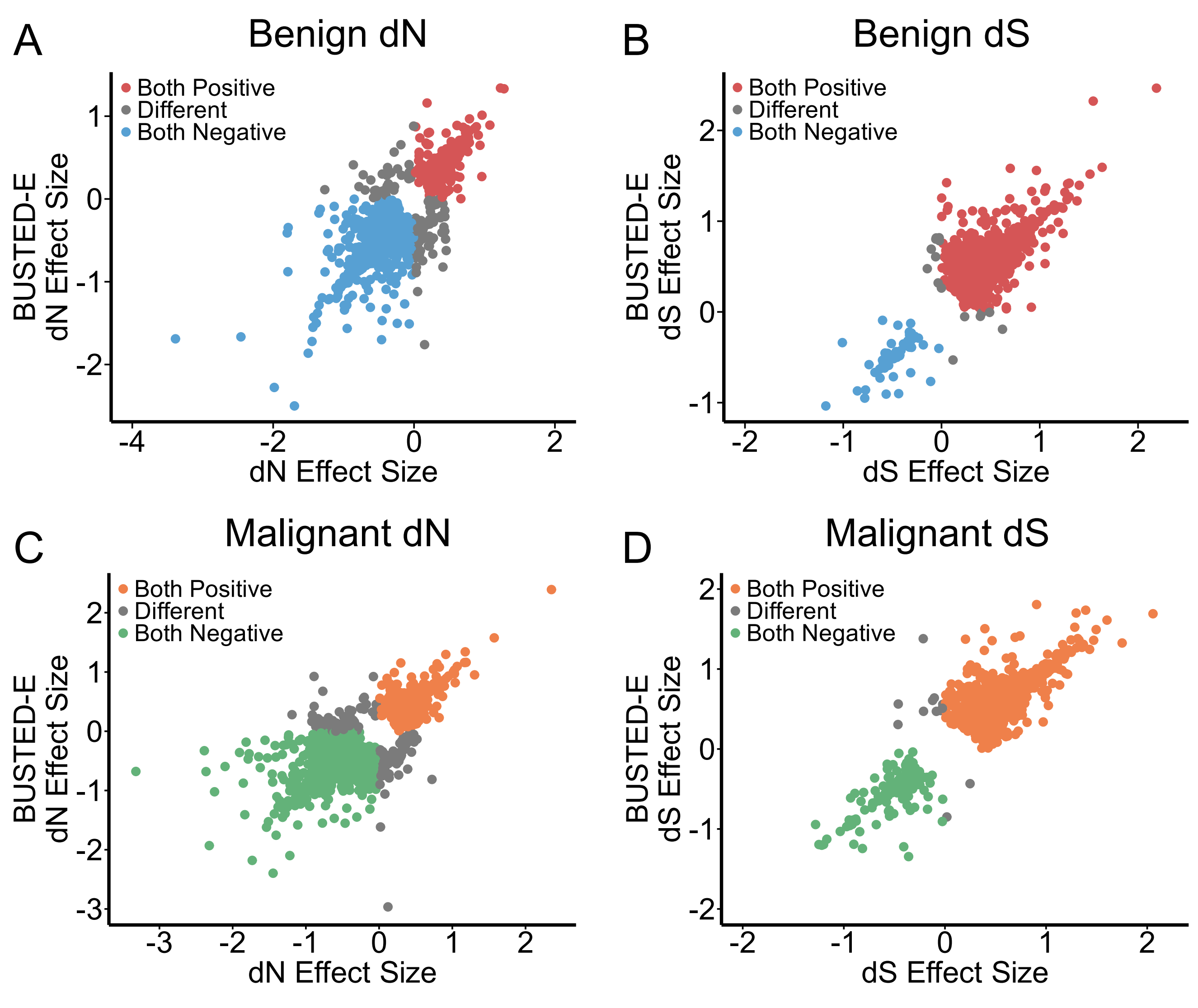
A
Benign dN
Both Positive
Different
Both Negative
BUSTED-E
dN Effect Size
dN Effect Size
B
Benign dS
Both Positive
Different
Both Negative
BUSTED-E
dS Effect Size
dS Effect Size
C
Malignant dN
Both Positive
Different
Both Negative
BUSTED-E
dN Effect Size
dN Effect Size
D
Malignant dS
Both Positive
Different
Both Negative
BUSTED-E
dS Effect Size
dS Effect Size

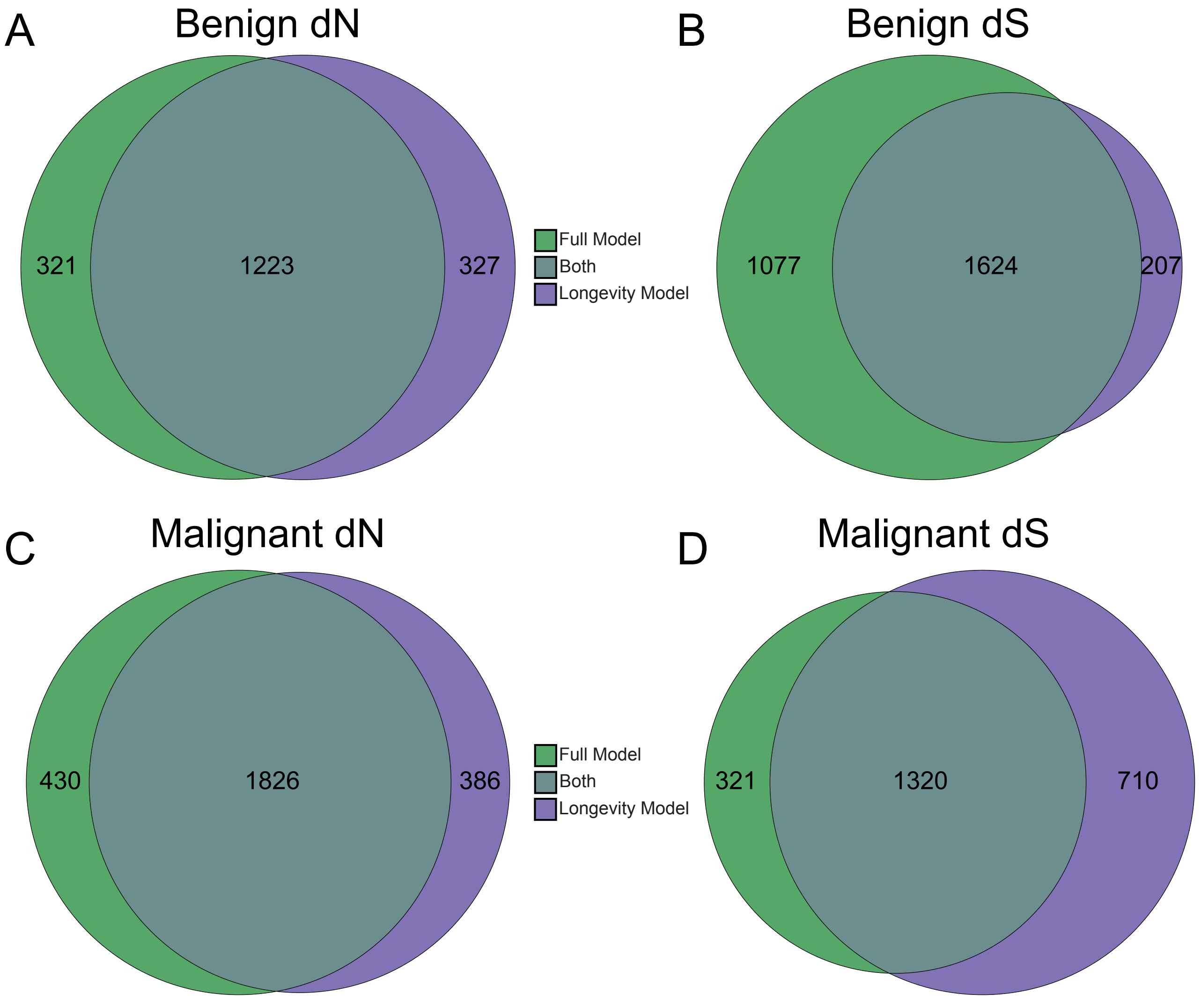
A
Benign dN
321
1223
327
Full Model
Both
Longevity Model
B
Benign dS
1077
1624
207
C
Malignant dN
430
1826
386
Full Model
Both
Longevity Model
D
Malignant dS
321
1320
710
